# Molecular surveillance confirms absence of avian influenza virus type A in Ecuadorian poultry: A retrospective reverse transcription quantitative polymerase chain reaction study from April 2023 to June 2024

**DOI:** 10.14202/vetworld.2025.2287-2294

**Published:** 2025-08-09

**Authors:** Byron Puga-Torres, Hugo Navarrete, David de la Torre

**Affiliations:** 1 Departamento de Biología Molecular, Laboratorio de Biología y Genética Molecular (LABIGEN), Quito, Ecuador; 2 Centro de Estudios Aplicados en Química (CESAQ), Pontificia Universidad Católica del Ecuador, Quito, Ecuador; 3 Institutional Review Boards, Institute for Research in Biological Sciences, Quito, Ecuador

**Keywords:** Avian influenza, Ecuador, H5N1, highly pathogenic avian influenza, One Health, Poultry, Reverse transcription quantitative polymerase chain reaction, surveillance

## Abstract

**Background and Aim:**

Avian influenza (AI) is a highly contagious zoonotic disease affecting birds and, occasionally, humans. Ecuador confirmed its first case of AI in late 2022, resulting in significant avian mortality and economic losses. In response, the Ecuadorian government implemented a mandatory control program emphasizing pre-vaccination diagnostics using reverse transcription quantitative polymerase chain reaction (RT-qPCR) or enzyme-linked immunosorbent assay. This study aimed to confirm the absence of AI virus type A in poultry across six major provinces of Ecuador following the 2022 outbreak, as part of the national eradication and vaccination campaign.

**Materials and Methods:**

A cross-sectional, retrospective molecular surveillance study was conducted from April 2023 to June 2024. A total of 343 pooled tracheal swab samples were collected from poultry farms in six provinces (Chimborazo, Cotopaxi, El Oro, Pastaza, Pichincha, and Tungurahua), covering over 1 million birds. The samples were analyzed using real-time RT-qPCR, targeting the M gene, and subtype-specific genes (H5, H7, and H7N9).

**Results:**

All 343 pooled samples (100%) tested negative for AI virus type A, suggesting an absence of active viral circulation during the study period. The implementation of pre-vaccination testing and biosecurity protocols contributed to this outcome.

**Conclusion:**

The study confirms that AI virus type A was not circulating in Ecuadorian poultry during the surveillance period. These findings underscore the effectiveness of collaborative efforts among government, industry, and laboratories. Ongoing molecular surveillance remains essential for early detection and prevention of future outbreaks, reinforcing Ecuador’s commitment to One Health principles.

## INTRODUCTION

Avian influenza (AI), also known as bird flu, is a highly contagious viral disease that affects both domestic and wild avian species. It is caused by influenza A viruses belonging to the *Orthomyxoviri-dae* family and is notable for its genetic variability and adaptability. These RNA viruses are spherical or pleomorphic in structure and are classified based on two surface glycoproteins – hemagglutinin (H) and neuraminidase (N) – which determine viral infectivity and host specificity. Among the influenza virus types (A, B, C, and D), only type A is zoonotic and capable of infecting a broad range of hosts, including birds, humans, horses, pigs, and dogs. Subtypes H5 and H7 are of particular concern due to their potential for high pathogenicity. The H5N1 subtype is especially lethal in birds and has caused severe illness in humans, including vulnerable populations such as pregnant women. Other subtypes such as H7N9, H9N2, and H6N1 are less pathogenic but remain zoonotic [1–8].

Wild birds, particularly waterfowl, serve as nat-ural reservoirs of AI viruses. Migratory species play a significant role in the transboundary spread of the virus. Key transmission risks include direct or indir-ect contact with infected birds, high-density housing conditions, poor biosecurity, inadequate sanitation, and improper disposal of infected carcasses. Environmental conditions such as cold temperatures and high humidity can also facilitate viral survival and dissemination. AI can manifest as either low pathogenic AI (LPAI), often asymptomatic or causing mild respiratory illness, or as highly pathogenic AI (HPAI), which results in severe systemic disease with mortality rates approaching 100%. The clinical outcome depends on the viral strain, host species, age, and immune status. In all cases, AI incurs substantial economic losses through depopulation eff-orts, trade restrictions, increased consumer costs, and biosecurity expenditures [4, 9–15].

Transmission of AI primarily occurs through horiz-ontal routes, including nasal, oral, ocular, and cloacal secretions. Indirect transmission through contaminated feed, water, aerosols, equipment, and personnel is also common. The fecal–oral route is considered the primary mode of transmission. AI viruses are environmentally resilient, capable of surviving for extended periods at low temperatures, enhancing their spread and persistence. Their genetic variability is driven by antigenic drift (point mutations in H or N genes) and antigenic shift (genetic reassortment occurs when multiple strains co-infect a host). While LPAI strains typically cause low mortality rates (<5%), HPAI viruses can result in mortality rates exceeding 90%, as seen with the H5N1 clade 2.3.4.4b, which caused widespread outbreaks in 2020–2021 [1, 3, 10, 11].

AI lacks pathognomonic clinical signs, making early detection challenging. The virus can replicate rapidly – within 24 h – and spread through the bloodstream or lymphatic system to target vital organs such as the heart, brain, and muscles. In HPAI infections, damage to the ciliated epithelium, immune cell dysfunction, and systemic inflammation contribute to rapid disease progression and high mortality. Lesions commonly include exudate plugs in the respiratory and digestive tracts, inflamed air sacs, and necrotic tissue. Mortality often occurs within days of symptom onset [1, 16, 17].

Diagnosis of AI requires both clinical observation and laboratory confirmation. Preferred samples include oropharyngeal and cloacal swabs, as well as tissues from organs such as the lungs, intestines, pancreas, and heart. Virus isolation in embryonated eggs remains the gold standard, followed by polymerase chain reaction (PCR) for subtype identification. Hemagglutination inhibition assays are used to identify H5 or H7 subtypes, and pathogenicity is determined using the intrav-enous pathogenicity index (IVPI); an IVPI >1.2 confirms high pathogenicity. Among molecular tools, real-time reverse transcription quantitative PCR (RT-qPCR) is widely regarded for its sensitivity and specificity in detecting viral RNA. It targets the conserved matrix (M) gene and the hemagglutinin (HA2) region, enabling the identification of all influenza A viruses and the specific detection of H5 and H7 subtypes [1, 10, 16, 18, 19].

RT-qPCR amplifies viral RNA with real-time fluorescence monitoring, allowing for qualitative and quantitative analysis. It reduces cross-contamination risks and provides rapid results essential for surveillance and outbreak response. Primers and TaqMan probes are designed to target conserved genetic regions, making RT-qPCR a robust diagnostic method even under conditions of pooled sampling or low viral loads [18, 19].

Globally, AI is a persistent threat due to the interplay between wild and domestic bird populations, international trade, and global migration pathways. Outbreaks have been reported across Asia, Europe, Africa, and the Americas. In Latin America, countries such as Mexico, Colombia, Peru, and Chile have experienced AI outbreaks, prompting the reinforcement of surveillance systems. In Ecuador, outbreaks reported since late 2022 have had a significant impact on poultry production and the rural livelihoods of its residents. Strengthening surveillance is therefore vital for early detection and control, and aligns with the One Health approach, which emphasizes the interdependence of human, animal, and environmental health [4, 9–12, 15, 20–23].

The World Organization for Animal Health (WOAH) mandates the reporting of AI cases and pro-vides international standards for disease control. In Ecuador, oversight is provided by the Ministry of Agriculture through Agency for Phytosanitary Regulation and Control (AGROCALIDAD), the national regulatory agency. AGROCALIDAD enforces mand-atory surveillance and vaccination protocols to prevent AI dissemination. Vaccination, when implemented in conjunction with robust diagnostics and biosecurity, has been demonstrated to be an effective strategy for reducing AI outbreaks worldwide [10, 20–23].

Despite the increasing prevalence and geographic spread of highly pathogenic AI (HPAI) worldwide, including in several Latin American countries, there is a notable lack of systematic molecular surveillance data from Ecuador following the country’s first confirmed outbreak in November 2022. While regional reports have documented the occurrence of AI in neighboring countries such as Colombia and Peru, peer-reviewed publications detailing the post-outbreak epidemiologi-cal landscape in Ecuador remain scarce. Moreover, few studies have addressed the effectiveness of government-mandated control strategies, including pre-vaccination RT-qPCR testing, in verifying the elimination of circulating influenza A viruses in domestic poultry populations. In this context, the scientific comm-unity and veterinary public health authorities require up-to-date molecular evidence to validate the current disease status in Ecuador and assess the ongoing risk to the poultry industry and public health. This absence of published surveillance data limits comparative analyses across the Andean region and hampers preparedness for future zoonotic threats within a One Health framework.

This study aimed to conduct a comprehensive, retrospective molecular surveillance of AI virus type A in poultry across six major Ecuadorian provinces between April 2023 and June 2024. Using real-time RT-qPCR, we analyzed tracheal swab samples collected from commercial farms as part of the national vaccination and disease control strategy mandated by AGROCALIDAD. By assessing over 300 pooled samples representing more than one million birds, this study aimed to verify the presence or absence of circulating AI virus type A post-outbreak and to evaluate the effectiveness of surveillance protocols and biosecurity interventions implemented nationwide. The results aim to inform policymakers, veterinarians, and epidemiologists on the current risk status, while contributing to regional and global One Health surveillance efforts.

## MATERIALS AND METHODS

### Ethical approval

The study was carried out in compliance with Ecuadorian regulatory standards, specifically Resolution 0021 issued by the AGROCALIDAD on March 2, 2023. The resolution mandated the nationwide vaccination of poultry flocks against highly pathogenic AI (HPAI), starting March 3, 2023. As a prerequisite for vaccination, each poultry farm was required to present a negative AI test result – either from enzyme-linked immunosorbent assay or RT-qPCR – conducted within 7 days before vaccination. These tests were to be performed exclusively by AGROCALIDAD laboratories or officially authorized laboratories within the national diagnostic network.

Due to the public health emergency and the mandatory nature of the surveillance, formal ethical approval was not sought. However, all procedures were carried out in accordance with national biosafety and animal welfare regulations. Personnel conducting sampling and testing followed appropriate biosafety protocols and used personal protective equipment to prevent zoonotic transmission.

### Study period and location

The surveillance period spanned from April 2023 to June 2024, coinciding with the enforcement of pre-vaccination diagnostic screening. Samples were obtained from commercial poultry farms located in provinces with the highest poultry production: Cotopaxi, Chimborazo, El Oro, Pastaza, Pichincha, and Tungurahua (Table 1).

**Table 1:** Description of the samples analyzed in this study.

Province	Region	Production line	Poultry farms(samples)	Number of total birds	Mean of brids	Age(weeks)	Type of sample	Type of test	Result
Chimborazo	Inter-Andean	Laying birds	38	881,079	23,813	Between 4 and 84	Tracheal swabs	RT-qPCR	Negative
Cotopaxi	Inter-Andean	Laying birds	126	4,417,174	35,912	Between 3 and 100	Tracheal swabs	RT-qPCR	Negative
Cotopaxi	Inter-Andean	Breeding birds	1	2,000	2,000	69	Tracheal swabs	RT-qPCR	Negative
El Oro	Coast	Laying birds	1	41,500	41,500	21	Tracheal swabs	RT-qPCR	Negative
Pastaza	Amazon	Laying birds	1	11,600	11,600	11	Tracheal swabs	RT-qPCR	Negative
Pichincha	Inter-Andean	Laying birds	11	484,500	69,214	Between 6 and 80	Tracheal swabs	RT-qPCR	Negative
Tungurahua	Inter-Andean	Laying birds	165	5,314,291	33,423	Between 1 and 104	Tracheal swabs	RT-qPCR	Negative

RT-qPCR=Reverse transcription quantitative polymerase chain reaction

### Study design

This observational, cross-sectional, and retros- pective study was conducted using laboratory data collected from poultry farms across six provinces in Ecuador.

### Sampling strategy and sample collection

To ensure representativeness and epidemiological coverage, farms selected for sampling were those requiring official AI testing to comply with vaccination guidelines. Tracheal swab samples were collected according to AGROCALIDAD protocols:

- Farms with ≥10,000 birds: 5 pooled samples
- Farms with <10,000 birds: 2 pooled samples.

Each pool consisted of swabs from five birds within a single poultry house. This pooling appr-oach, commonly used in low-prevalence surveillance programs, enhances testing efficiency, reduces costs, and maintains sufficient sensitivity when paired with RT-qPCR.

In total, 343 pooled samples were collected, repre-senting approximately 1,115,214 birds aged between 1 and 104 weeks (Table 1). These samples were selected based on farm-level submissions and inclusion crite-ria and were processed by Laboratorio de Biología y Genética Molecular (LABIGEN), an officially authorized diagnostic laboratory based in Quito, Ecuador.

### Inclusion and exclusion criteria

Samples were included if they met the following criteria:

- Originated from poultry farms in Ecuador
- Sampled between April 2023 and June 2024
- Tested using RT-qPCR for AI Type A
- Included metadata on province/canton, production line (laying or breeder), number and age of birds, and test result.

Records lacking complete metadata or test type were excluded from the study. Only domestic poultry were included in this study; wild birds were not surveyed.

These criteria ensured a robust, epidemio-logically valid dataset to support decision-making, risk assessment, and disease control efforts. Additionally, these parameters enable future analyses of outbreak distribution by production system, flock size, or bird age.

### Laboratory procedures (RT-qPCR)

All laboratory analyses were performed at LABIGEN using real-time RT-qPCR (Table 2). The diagn-ostic protocol was based on methods described by Spackman *et al*. [24] and the WOAH protocol for dete-cting H7N9 [25].

**Table 2:** PCR primer and hydrolysis probe sequences of AI virus.

Specificity	Primer/Probe	Sequence(5×–3×)
Avian influenza virus	M+25	AGA TGA GTC TTC TAA CCG AGG TCG
	M-124	TGC AAA AAC ATC TTC AAG TCT CTG
	M+64	FAM-TCA GGC CCC CTC AAA GCC GA-TAMRA

FAM=6-carboxyfluorescein, TAMRA=6-carboxytetramethylrhodamin, PCR=Polymerase chain reaction, AI=Avian influenza

### Equipment and reagents

- Thermal cycler: QuantStudio 3 (Applied Biosystems, USA)
- Universa RNA extraction kit: (Nanjing Vazyme Medical Technological Industry, Nanjing-China)
- Cycle threshold cutoff value: 35
- Positive control RNA: Provided by AGROCALIDAD.

### RT-qPCR workflow

1. Viral RNA extraction: RNA was isolated from pooled tracheal swabs using commercial RNA extraction kits.
2. One-step RT-qPCR: Reverse transcription and amplification were conducted in a single-tube format using thermostable reverse transcriptase and Taq DNA polymerase.
3. Primers and probes: TaqMan probes targeting the conserved matrix (M) gene and subtype-specific hemagglutinin (HA) genes (H5, H7, and H7N9) were used.
4. Cycling conditions and detection: A standard cycling protocol was applied (reverse transcription at ~50°C, initial denaturation at ~95°C, followed by 40 cycles). Fluorescence signals were recorded in real-time to determine presence or absence of viral RNA (Table 2).

### Statistical analysis

All laboratory results and associated metadata were entered into Microsoft Excel version 2021 (Microsoft, Washington, USA) and screened for completeness and consistency. Frequency tables were used to describe the distribution of samples by province, production type, flock size, and bird age. Statistical analyses were performed using R (RStudio v2024.04.2+764, R Core Team, Vienna, Austria), with a focus on descriptive epidemiology.

Although no positive AI samples were identified, a rigorous data validation process was conducted, including the elimination of duplicates and the correction of inconsistencies. Due to the absence of detected cases, inferential statistics could not be applied.

## RESULTS

### Sample characteristics

Between April 2023 and June 2024, a total of 343 pooled tracheal swab samples were collected and analyzed from poultry farms across six Ecuadorian provinces: Chimborazo, Cotopaxi, El Oro, Pastaza, Pichincha, and Tungurahua. Of these, 342 samples (99.71%) originated from laying hens, while only one sample (0.29%) was collected from breeder flocks. The flocks varied widely in size, ranging from 5 to 600,000 birds, and included birds aged between 1 and 130 weeks.

## RT-qPCR results

All 343 samples tested negative for AI virus Type A using real-time RT-qPCR (Table 2). This result suggests that there was no active virus circulation during the surveillance period in the sampled regions of Ecuador. The findings reflect the effectiveness of the coordin-ated efforts undertaken by the Ecuadorian government, private poultry producers, and authorized laborato-ries to prevent disease resurgence following the 2022 outbreak.

### Considerations on diagnostic reliability

Although RT-qPCR is considered the gold standard for AI detection due to its high sensitivity and specificity, certain factors can contribute to false-negative results. These include suboptimal sample quality, low viral load, viral mutations in primer/probe regions, or the pres-ence of PCR inhibitors. As such, continuous monitoring remains essential to ensure ongoing disease-free status.

### Limitations in breeder flock representation

It is important to note that only one sample (1/343) was obtained from breeder flocks. This limited representation may restrict the generalizability of findings to this specific poultry population. Fut-ure surveillance efforts should consider increasing breeder flock sampling to strengthen epidemiological conclusions for this group.

## DISCUSSION

### One Health success in combating AI in Ecuador

The confirmed absence of AI virus circulation during the surveillance period represents a significant achievement for Ecuador’s One Health strategy. The coordinated efforts of government authorities, private industry, and diagnostic laboratories have contributed to reducing the zoonotic risk posed by AI, particularly the highly pathogenic subtypes such as H5 and H7.

### Importance of rapid diagnosis and biosecurity measures

Early detection and rapid containment of AI out-breaks depend on accurate diagnostics and timely surveillance. Serological and molecular testing play a central role in monitoring disease status. Infected flocks must be promptly depopulated, and the safe disposal of carcasses, eggs, and manure must follow biosafety protocols to prevent environmental contamination. In the case of HPAI, depopulation and disposal typically involve incineration or composting. For LPAI, recove-red birds may be sold under controlled conditions, and eggs can be marketed following appropriate disinfection measures [17, 20, 26, 27].

### Global impact and human zoonotic potential

Between 2005 and 2021, HPAI outbreaks resulted in the death or culling of over 316 million commercial birds globally, with major peaks in 2006, 2016, 2017, and 2021. These outbreaks occurred in over 50 countries and have included occasional zoonotic spillover events.

- H5N1 has caused ~870 human infections, with a mortality rate of ~50%.
- H5N6 and H9N2 have caused ~80 human infections each, with 30 and 20 deaths, respectively.
- Other subtypes such as H3N8, H7N4, H7N7, and H10N3 have also been sporadically reported, underscoring the ongoing zoonotic threat [28].

### Recent global spread of HPAI (2021–2023)

According to the WOAH [10], between April 21 and May 4, 2023, 12 AI outbreaks occurred in comme-rcial and backyard poultry in Europe, the Americas, and Africa, resulting in the loss of approximately 300,000 birds through culling or disease. Since 2021, migratory bird pathways have facilitated the spread of AI across continents, affecting wild and domestic bird populations in Canada, the United States, and Mexico. By October 2022, outbreaks had spread to South America, affecting countries such as Colombia, Ecuador, Peru, Venezuela, and Chile.

### Country-level examples of control and spread

China has faced repeated and severe outbreaks, including H5N1 and H5N8, requiring trade restrictions and mass culling operations [29]. In the United Kingdom, the high wild bird migration in 2021–2022 triggered government-imposed control measures, including cull-ing and restrictions on the movement of production farms [30].

### Latin American context and economic consequences

AI has posed considerable challenges in Latin America, particularly for poultry-producing countries.

- Brazil, one of the world’s largest meat producers, experienced widespread outbreaks of H5N1 in 2023 across its southern and central-western regions, resulting in significant economic losses [31].
- Argentina and Chile reported isolated cases with limited economic impact.
- Ecuador, Peru, and Colombia reported more widespread outbreaks, prompting quarantines and heightened biosecurity measures to protect national food security and agricultural trade [32].

### Ecuador’s 2022 outbreak and spillover to human

Ecuador confirmed its first outbreak of AI on November 29, 2022. The Ministry of Agriculture and AGROCALIDAD reported that an initial outbreak affecting 350,000 birds was followed by further infections in an additional 500,000 birds, with 17,000 more confir-med cases in later outbreaks. Most new cases were concentrated in Cotopaxi and Bolívar provinces [21].

Subsequently, on January 09, 2023, the first human case of H5 AI in Latin America and the Caribbean was confirmed in Ecuador. The case was detected thro-ugh sentinel surveillance for severe acute respiratory infections and involved a 9-year-old girl who had direct contact with infected birds. This infection was validated by the National Institute of Public Health Research and reported to the World Health Organization [11, 20].

### The need for regional surveillance collaboration

Given Ecuador’s geographic proximity to Colombia and Peru, and the known migratory pathways of wild birds between these countries, regional cooperation in AI surveillance is essential. Monitoring programs must extend beyond domestic poultry to include wild birds and susceptible mammalian species. This approach will facilitate early detection, minimize zoonotic risk, and protect both public health and food systems.

Examples from the region support this strategy:

- In Colombia (2010–2012), surveillance in the Los Llanos region identified low-pathogenic AI strains (e.g., H5N2) in wild and domestic birds [33].
- In Peru (2016), researchers documented extensive genetic diversity among influenza A strains in wild birds [34].
- In 2023, clade 2.3.4.4b H5N1 was identified in wild birds, poultry, and marine mammals in the Pantanos de Villa reserve near Lima [35].

### Contribution to regional One Health surveillance

This study addresses a critical gap in the literature on AI surveillance in South America and Ecuador. By providing molecular evidence of AI absence during a high-risk period, it supports Ecuador’s One Health policy and offers a framework for surveillance-based disease control. Continuous, coordinated surveillance is impe-rative to safeguard animal health, protect the poultry industry, and mitigate zoonotic risks.

## CONCLUSION

This retrospective molecular surveillance study analyzed 343 pooled tracheal swab samples collected from poultry farms across six major provinces in Ecuador between April 2023 and June 2024. All samples tested negative for AI virus type A using RT-qPCR, indicating no evidence of active viral circulation in the sampled regions during the surveillance period. The dataset covered over one million birds, primarily laying hens, with flock ages ranging from 1 to 130 weeks. These findings highlight the effectiveness of Ecuador’s national AI control program, which incorporates compulsory pre-vaccination testing, regulated vaccine deployment, and stringent biosecurity protocols. The absence of AI supports the safe continuation of poultry production and export, while also reducing the risk of zoonotic diseases to humans. The results also serve as critical evidence for maintaining Ecuador’s AI-free status in international trade frameworks.

This study is the first to provide large-scale, laboratory-confirmed evidence of AI absence in Ecuador following the 2022 outbreak. It utilized a standardi-zed and internationally recognized diagnostic protocol (RT-qPCR) across geographically diverse provinces, supporting national One Health objectives by bridging the veterinary and public health domains. However, breeder flocks were underrepresented, with only one sample collected, which limits the generalizability to this important production group. Wild bird populat-ions, which may act as reservoirs or vectors, were not included in this surveillance. As a cross-sectional design, the study reflects a specific time frame and may not capture future or intermittent viral incursions.

Future surveillance efforts should aim to increase sampling from breeder flocks and backyard poultry operations, incorporate surveillance of wild birds and mammalian wildlife – particularly in migratory corridors – and establish a longitudinal monitoring framework to detect viral introductions in real-time and assess vaccine efficacy under field conditions. The confirmed absence of AI virus type A in Ecuadorian poultry from April 2023 to June 2024 reflects the success of coor-dinated national control efforts. Sustained molecular surveillance, expanded ecological monitoring, and regional collaboration will be vital to maintaining this disease-free status. These results contribute valuable data to the regional AI control agenda and exemplify the practical implementation of One Health principles in South America.

## AUTHORS’ CONTRIBUTIONS

BP and DT: Conception and design of the study. DT: Laboratory analysis. BP and DT: Conducted the study and analyzed and interpreted the data. BP, DT, and HN: Conducted the assessment and interpreted the results. BP: Drafted the manuscript. DT and HN: Critically reviewed and revised the manuscript. All authors have read and approved the final manuscript.
